# Study on the Depression Performance and Mechanism of the Novel Chalcopyrite Depressant 2-Mercapto-5-benzimidazole Sulfonate Dihydrate in the Flotation Separation of Cu-Mo Bulk Concentrate

**DOI:** 10.3390/molecules31132383

**Published:** 2026-07-06

**Authors:** Jianhua Chen, Xufu Zhang, Lujing Liang, Anruo Luo

**Affiliations:** 1 School of Resources, Environment and Materials, Guangxi University, Nanning 530004, China; jhchen@gxu.edu.cn (J.C.); 15778638817@163.com (L.L.); luoanruo123@163.com (A.L.); 2 Yunnan Diqing Nonferrous Metals Co., Ltd., Xianggelila 674499, China

**Keywords:** depressant, chemisorption chalcopyrite, Cu–Mo separation, molecular design, adsorption mechanism

## Abstract

Chalcopyrite and molybdenite exhibit similar surface wettability and high floatability, which has long hindered their efficient and selective separation in mineral processing. In this work, the novel chalcopyrite depressant 2-mercapto-5-benzoimidazole sulfonate dihydrate (2MBI5SA) was investigated for its effect on the flotation behavior of chalcopyrite and molybdenite. Compared with the conventional depressant sodium sulfide (Na_2_S), 2MBI5SA exhibited stronger selective depression toward chalcopyrite; under conditions yielding a Mo recovery of 81.46% and a Mo grade of 4.46%, the Cu recovery decreased to 13.03%. To clarify the origin of this selectivity, interfacial properties were systematically characterized using adsorption measurements, contact angle measurements, zeta potential measurements, FTIR, XPS, and SEM-EDS, and the adsorption mechanism was further elucidated using SCC-DFTB calculations. The results demonstrate that 2MBI5SA chemisorbs onto the chalcopyrite surface via bidentate coordination, forming a stable adsorption layer that effectively suppresses chalcopyrite flotation. Moreover, structure–function relationship analysis confirmed that introducing hydrophilic and ionizable functional groups into the collector framework can convert a collector into a selective depressant, thereby providing new insights into the rational design of selective organic depressants with potential environmental advantages over conventional highly toxic inorganic depressants.

## 1. Introduction

Molybdenum is a critical material for power electronics, advanced manufacturing, aerospace, chemical catalysis, and high-end alloys, making its stable supply and efficient utilization strategically important [1,2,3]. Approximately 75% of global copper resources [4,5] and 50% of molybdenum resources [6] are associated with Cu–Mo-associated ores [7], which are predominantly hosted in porphyry Cu–Mo sulfide deposits [8]. Because chalcopyrite (CuFeS_2_) and molybdenite (MoS_2_) commonly occur together and exhibit similar natural floatability [9], their flotation separation is challenging and has long limited efficient resource recovery. Therefore, advancing the theoretical understanding and process technology for Cu–Mo separation is important for improving resource utilization efficiency.

In industrial practice, flotation separation of Cu–Mo bulk concentrates typically follows a strategy of depress chalcopyrite while floating molybdenite [10,11], the core of which lies in developing highly selective chalcopyrite depressants [12,13]. An ideal depressant should weaken the interaction between chalcopyrite and the collector while enhancing the hydrophilicity of the mineral surface, thereby amplifying the interfacial differences between chalcopyrite and molybdenite to achieve effective separation [14]. Traditionally, inorganic depressants have been the most mature class of chalcopyrite depressants, and owing to their wide availability, low cost, and ease of integration into flotation circuits, they remain widely used in many mineral processing plants. These inorganic reagents mainly include sulfide-based depressants [15,16,17], cyanides, Knox-type reagents [12,18], oxidants [19,20,21,22,23,24,25], and precipitation–coverage-type depressants [24,26,27,28]. However, with increasing demands for environmental protection and safety, the limitations of inorganic depressants, such as insufficient selectivity, high dosage requirements, and significant environmental impact, have become increasingly apparent. In particular, the industrial use of cyanide and Knox-type reagents has declined due to their high toxicity and associated pollution risks.

In contrast, organic depressants have demonstrated significant advantages in selectivity and tunability, and are therefore regarded as a promising alternative to traditional inorganic depressants for achieving environmentally benign Cu–Mo separation. The structures of such organic molecules typically consist of a surface-affinitive moiety and a hydrophilic group [29,30,31,32], and their major structural motifs include thiourea-based [33,34,35,36,37,38], mercaptan-based [39,40,41,42,43,44,45,46], carbonyl-based [47,48,49,50,51], hydroxyl-based [52], and xanthate-based depressants [53]. Among them, mercaptan-containing (–SH) organic depressants are of particular interest due to their relatively low toxicity, favorable environmental compatibility, and strong propensity to adsorb onto surface metal active sites, factors that have been shown to facilitate selective separation of chalcopyrite and molybdenite in multiple studies [41,43,44,54,55]. However, current screening of depressant molecules still largely relies on the empirical trial-and-error approach, and the structure–function relationships and rational design strategies remain unclear, thereby limiting the predictive design of new depressants.

Previous studies have demonstrated that molecular design is an effective strategy for regulating the structure–function relationship of flotation reagents. For collectors, adjustment of the molecular hydrophilic/hydrophobic balance and the introduction of oxygen atoms, alkoxyl groups, hydroxyl groups, or other functional moieties can markedly influence surface activity, selective adsorption, frothing behavior, and collecting performance. For example, the introduction of alkoxyl groups into ether thionocarbamate collectors has been reported to improve their affinity toward chalcopyrite and frothing properties. Hydroxyl-containing thionocarbamate collectors have also been shown to affect chalcopyrite flotation through the synergistic effect between the hydroxyl group and the collecting group. In addition, 2-mercaptobenzimidazole (2MBI) and its derivatives have been reported as chelating collectors for sulfide minerals, indicating that the mercapto group and heterocyclic nitrogen atoms in this molecular skeleton are capable of interacting with surface metal sites. These studies indicate that functional-group regulation has a clear basis in the design of flotation reagents. However, previous studies have mainly focused on enhancing the collecting ability, selectivity, or frothing properties of collectors through structural optimization, or on directly screening new organic depressants. The transformation of a 2MBI-type collector skeleton into a selective chalcopyrite depressant for Cu–Mo separation through the introduction of a strongly hydrophilic and ionizable functional group has not been systematically clarified. Therefore, in this work, a sulfonate group was introduced into the 2MBI-type skeleton to develop a novel chalcopyrite depressant with both selective surface-coordination ability and surface-hydrophilization regulation ability. Building on these considerations, we propose a novel design strategy in which introducing hydrophilic and ionizable functional groups into a collector framework can convert such molecules from collectors into selective depressants toward target minerals. To validate this strategy, 2MBI was chosen as the parent compound and modified by incorporating a sulfonate group to form 2-mercapto-5-benzimidazole sulfonate dihydrate (2MBI5SA), which was then investigated as a chalcopyrite depressant for Cu–Mo separation. We hypothesize that 2MBI5SA operates through a synergistic mechanism: its mercapto group and benzimidazole nitrogen atoms serve as cooperative coordination sites that strongly chemisorb onto metal active sites on the chalcopyrite surface to form stable chelate complex; meanwhile, its sulfonate group [56,57] enhances hydration and electrostatic repulsion, thereby suppressing the floatability of chalcopyrite.

To elucidate the surface interaction mechanisms underlying the observed behavior, this study systematically characterized interfacial properties including adsorption measurements, contact angle measurements, zeta potential measurements, FTIR, XPS, and SEM-EDS, and further elucidated the adsorption process using SCC-DFTB simulations. The results demonstrate that 2MBI5SA chemisorbs onto the chalcopyrite surface through a bidentate coordination mode, forming a stable surface adsorption layer that substantially alters the surface electronic structure and wettability. Structure–function relationship analysis further confirms that introducing hydrophilic and ionizable functional groups into a collector framework can convert its role from facilitating flotation to serving as a selective depressant. These findings contribute to a deeper understanding of surface adsorption chemistry in flotation systems and provide guidance for the rational design of selective organic flotation depressants.

## 2. Results and Discussion

### 2.1. Single-Mineral Microflotation Tests

**Figure 1:**
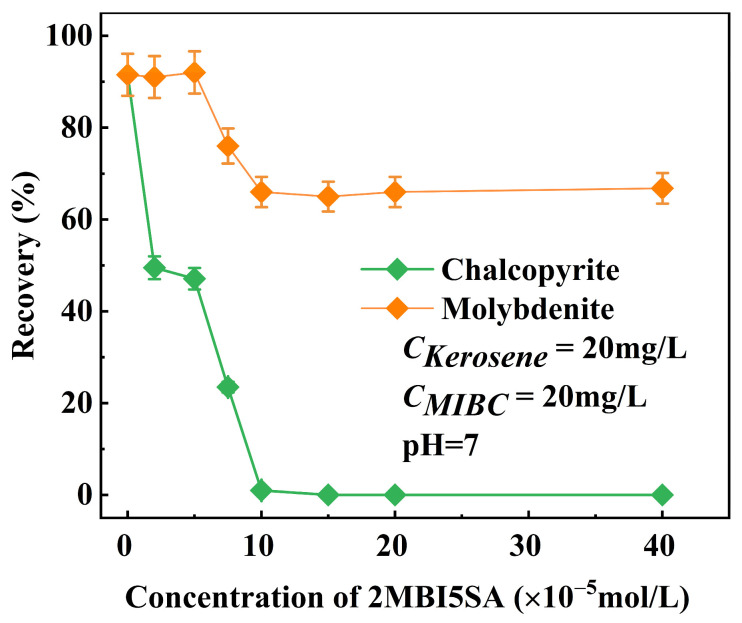
Effect of 2MBI5SA dosage on the flotation performance of chalcopyrite and molybdenite in single-mineral microflotation tests.

Figure 1 illustrates the influence of 2MBI5SA dosage on the flotation behavior of chalcopyrite and molybdenite using kerosene as the collector at pH 7. The results demonstrate that 2MBI5SA exerts a strong selective depression toward chalcopyrite. Specifically, chalcopyrite recovery decreased sharply with increasing 2MBI5SA dosage, and when the dosage reached 1 × 10^−4^ mol/L, the chalcopyrite recovery declined to 1%, indicating excellent depressing performance; further increases in dosage did not lead to significant additional changes in recovery. In contrast, increasing the 2MBI5SA dosage resulted in only a slight decrease in molybdenite recovery, which remained above 66.0% throughout the tested range. In contrast, molybdenite recovery showed a moderate decrease at higher 2MBI5SA dosages, although it remained above 66.0% throughout the tested range. This decrease suggests that excessive 2MBI5SA may also exert a weak adverse effect on molybdenite flotation, probably because a small amount of 2MBI5SA can adsorb on molybdenite edge sites or surface defects, and the hydrophilic sulfonate group may partially hinder kerosene adsorption or spreading and bubble–particle attachment. Nevertheless, the decrease in chalcopyrite recovery was much more pronounced than that of molybdenite, indicating that 2MBI5SA still exhibits preferential depression toward chalcopyrite within an appropriate dosage range.

For comparison, 10 × 10^−5^ mol/L 2MBI5SA in the single-mineral tests corresponds to approximately 0.53 kg/t, which is lower than the dosage used in the actual Cu–Mo bulk concentrate tests; this difference is reasonable because the real ore system is more complex and may consume more reagent.

**Figure 2:**
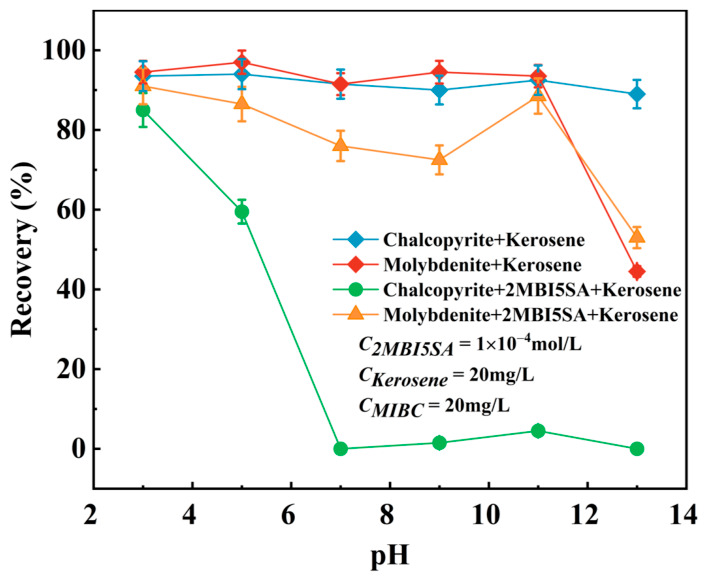
Effect of 2MBI5SA on the floatability of chalcopyrite and molybdenite at different pH values in single-mineral microflotation tests.

Figure 2 shows the effect of slurry pH on the flotation behavior of chalcopyrite and molybdenite at a fixed 2MBI5SA concentration of 1 × 10^−4^ mol/L, together with the results obtained in the absence of depressant. Without 2MBI5SA, chalcopyrite maintained a high recovery (90%) across a wide pH range of 3–13, indicating that its inherent floatability was only slightly affected by pH. In contrast, molybdenite recovery remained high in the pH range of 3–7 but decreased markedly at pH 11 and above. This decrease was not considered to be an experimental anomaly, because all microflotation tests were performed in triplicate and the averaged values were reported. Instead, it may be related to the intrinsic pH-dependent surface chemistry of molybdenite under strongly alkaline conditions: enhanced surface deprotonation or OH^−^ adsorption and possible oxidation or hydration of edge sites can increase the surface hydrophilicity of molybdenite, thereby weakening kerosene adsorption or spreading and bubble–particle attachment. Upon addition of 2MBI5SA, the flotation behaviors of the two minerals diverged significantly. Chalcopyrite recovery decreased sharply with increasing pH, dropping to as low as 0.3%, and then remained nearly constant at pH 7 and above. This trend may be attributed to progressive deprotonation of the sulfonic acid group (–SO_3_H to SO_3_^−^) at higher pH, which increases the negative charge and hydrophilicity of 2MBI5SA, thereby rendering the chalcopyrite surface more hydrophilic and more effectively suppressing the adsorption and spreading of kerosene. In contrast, molybdenite maintained relatively high recovery under 2MBI5SA treatment. Overall, a pH range of 7–13 was favorable for the efficient separation of chalcopyrite and molybdenite.

### 2.2. Flotation Tests of a Synthetic Mineral Mixture

**Figure 3:**
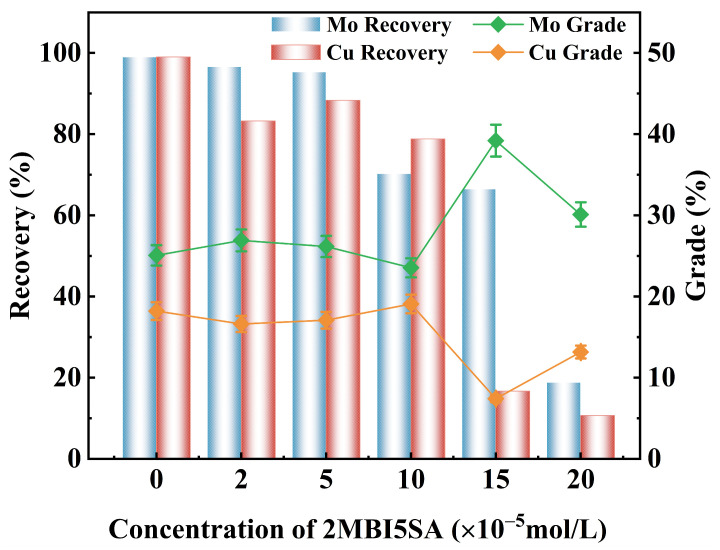
Flotation recoveries and grades of Cu and Mo in synthetic mixed-mineral flotation tests under different 2MBI5SA dosages. The mass ratio of chalcopyrite to molybdenite was 1:1, and the tests were conducted at pH 7 with 20 mg/L kerosene.

Single-mineral flotation tests confirmed the strong depressing effect of 2MBI5SA on chalcopyrite. Figure 3 presents the effect of 2MBI5SA dosage on the flotation behavior of synthetic mineral mixture composed of chalcopyrite and molybdenite at a mass ratio of 1:1. In the absence of a depressant, the Mo and Cu recoveries in the concentrate were 99.01% and 99.02%, respectively, indicating that the two minerals could not be effectively separated. With increasing 2MBI5SA dosage, molybdenite recovery remained high initially and then exhibited a gradual decline, whereas Cu recovery decreased progressively with increased dosage. At a dosage of 15 × 10^−5^ mol/L, Mo recovery and grade reached 66.47% and 39.2%, respectively, while Cu recovery and grade were 16.68% and 7.42%, respectively. Further increasing the dosage led to higher molybdenite grade but eventual molybdenite loss due to over-dosage. It is noteworthy that, compared with single-mineral tests, synthetic mixed minerals flotation required a higher 2MBI5SA dosage to effectively suppress chalcopyrite.

### 2.3. Industrial Ore Flotation Tests

**Figure 4:**
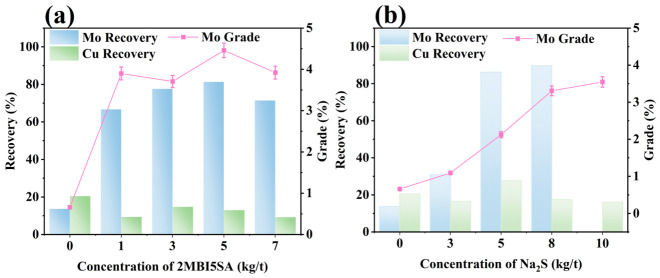
Comparison of 2MBI5SA and Na_2_S in the flotation separation of actual Cu–Mo bulk concentrate: (**a**) 2MBI5SA; (**b**) Na_2_S.

To further validate the selective depression of chalcopyrite by 2MBI5SA under industrial conditions, flotation tests were conducted on actual Cu–Mo bulk concentrate. Figure 4 compares the flotation separation performance of 2MBI5SA with that of the conventional depressant sodium sulfide (Na_2_S). Under the 2MBI5SA system, Mo recovery and grade increased with increasing dosage, reaching a maximum of 81.41% and 4.46%, respectively, at 5 kg/t. However, further increases in dosage resulted in declines in both Mo recovery and grade, indicating that excessive addition was detrimental to separation indices. In the Na_2_S system, Mo recovery and grade also increased with dosage, but Cu recovery remained generally higher than in the 2MBI5SA system, suggesting that Na_2_S, while enhancing Mo recovery, more readily promoted non-selective Cu flotation, thereby limiting concentrate grade. Importantly, 2MBI5SA demonstrated stronger separation performance at lower dosages: at 1 kg/t, Mo recovery and grade reached 66.75% and 3.90%, respectively, significantly outperforming the Na_2_S system at 3 kg/t, where the respective values were only 30.98% and 1.09%. These results suggest that 2MBI5SA offers a clear dosage advantage, achieving more effective molybdenite enrichment and recovery at lower addition levels.

### 2.4. Adsorption Capacity Results

**Figure 5:**
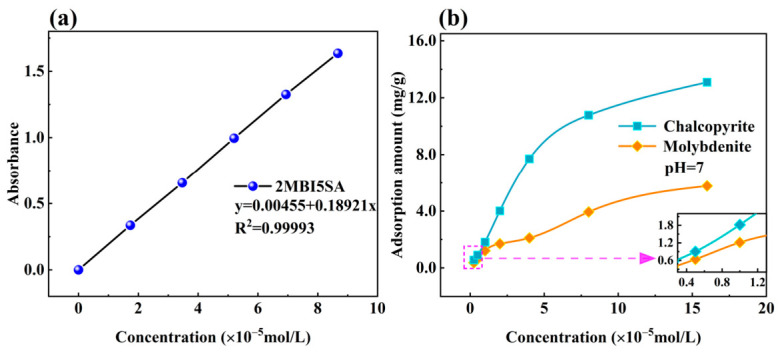
(**a**) Calibration curve of 2MBI5SA at different concentrations; (**b**) adsorption amounts of 2MBI5SA on chalcopyrite and molybdenite.

The calibration curve of 2MBI5SA constructed over a range of concentrations (Figure 5) shows an R^2^ value of 0.99993, indicating that the UV-Vis spectrophotometric method is reliable. Figure 5b shows the relationship between the initial 2MBI5SA concentration and the amount on chalcopyrite and molybdenite surfaces at pH 7. With increasing initial concentration, the adsorption amounts on both minerals increased monotonically. At an initial concentration of 1 × 10^−4^ mol/L, the adsorption amounts of 2MBI5SA on the chalcopyrite and molybdenite were 13.09 mg/g and 5.77 mg/g, respectively. These results demonstrate that 2MBI5SA preferentially adsorbs onto chalcopyrite, exhibiting higher selectivity than for molybdenite.

### 2.5. Contact Angle Measurements

**Figure 6:**
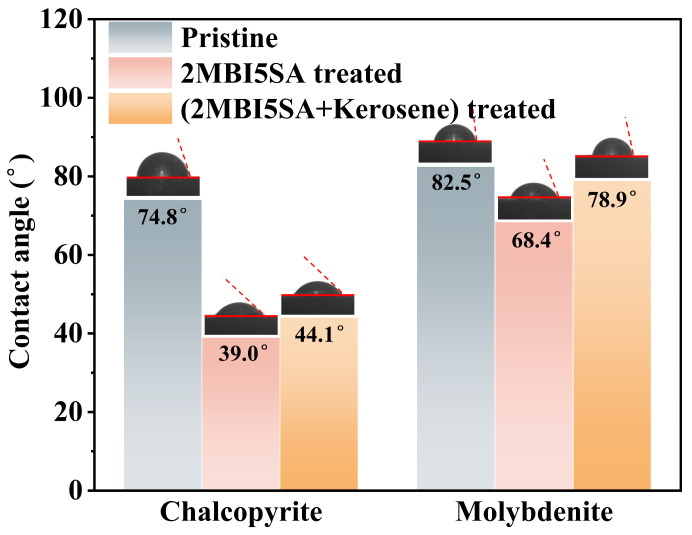
Contact angles of chalcopyrite and molybdenite under different treatment conditions (Pristine, 2MBI5SA-treated, and (2MBI5SA + Kerosene)-treated).

Contact angle is an important parameter for evaluating the wettability of minerals [58]. In general, a smaller contact angle indicates higher hydrophilicity and lower floatability, whereas a larger contact angle implies stronger hydrophobicity and higher floatability. Figure 6 shows the contact angles of chalcopyrite and molybdenite under different reagent-treatment conditions to evaluate the effect of 2MBI5SA on surface wettability. Untreated chalcopyrite and molybdenite exhibited contact angles of 74.8° and 82.5°, respectively, indicating relatively hydrophobic surfaces and higher floatability for both minerals. After conditioning with 2MBI5SA, the contact angle of chalcopyrite decreased markedly to 39.0°, indicating a shift toward a much more hydrophilic surface; by contrast, the contact angle of molybdenite decreased to 68.4°. After combined treatment with 2MBI5SA and kerosene, the contact angle of chalcopyrite increased slightly to 44.1°, suggesting that the hydrophilic induced by 2MBI5SA largely persists and suppresses the restoration of hydrophobicity by kerosene on chalcopyrite. In contrast, the contact angle of molybdenite increased to 78.9°, indicating substantial adsorption of kerosene on molybdenite. Overall, these results indicates that 2MBI5SA amplifies the wettability contrast between chalcopyrite and molybdenite, which is favorable for selective flotation separation.

### 2.6. Zeta Potential Analysis

**Figure 7:**
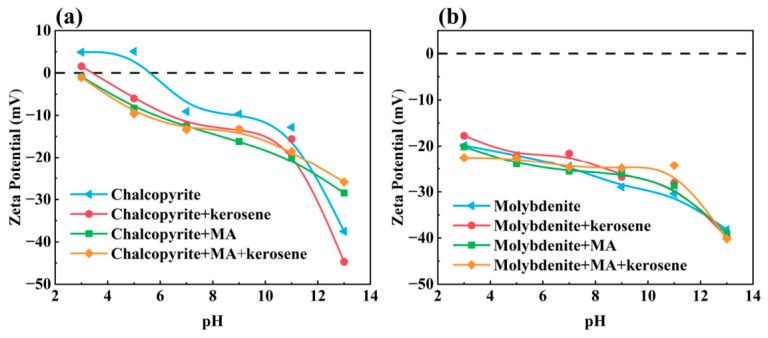
Zeta potential versus pH curves for chalcopyrite (**a**) and molybdenite (**b**) under different conditions. (2MBI5SA 10 × 10^−5^ mol/L, Kerosene 20 mg/L).

Zeta potential is widely used to assess the surface charge state of mineral particles and their interfacial behavior during flotation, providing important insights into reagent adsorption mechanisms [59]. Figure 7 shows the variation in zeta potential as a function of pH for chalcopyrite and molybdenite under different treatment conditions. For both minerals, the zeta potentials became more negative with increasing pH, which can be mainly attributed to enhanced surface deprotonation and adsorption of OH^−^. Chalcopyrite exhibited an isoelectric point at approximately pH 4.0, whereas molybdenite remained negatively charged over the examined pH range. After conditioning with 2MBI5SA, both chalcopyrite and molybdenite showed negative shifts in zeta potential, indicating that the ionizable sulfonate-containing reagent can modify the surface charge of both minerals to some extent. It should be noted that the absolute zeta-potential values of the two minerals after 2MBI5SA treatment were relatively close at pH 7; therefore, the zeta-potential results alone cannot fully explain the flotation selectivity between chalcopyrite and molybdenite. Instead, these results should be interpreted as supporting evidence for surface charge modification after reagent adsorption.

### 2.7. FTIR Analysis

**Figure 8:**
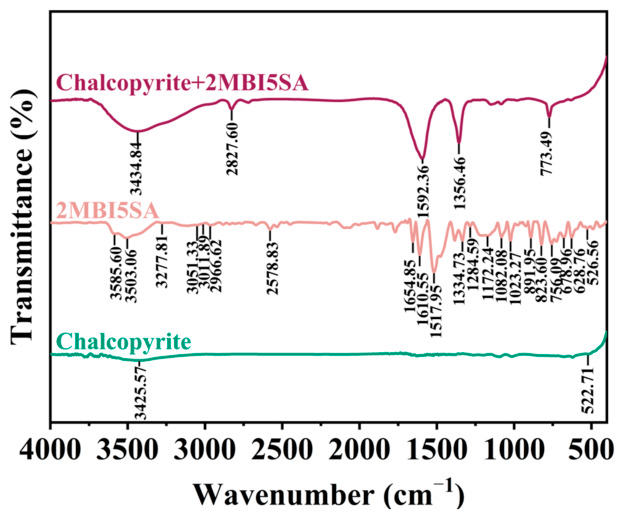
FTIR spectra of 2MBI5SA, 2MBI5SA-treated chalcopyrite, and untreated chalcopyrite samples.

Fourier transform infrared spectroscopy (FTIR) is widely employed to probe the adsorption of flotation reagents on mineral surfaces. Figure 8 shows the FTIR spectra of 2MBI5SA and chalcopyrite before and after conditioning with 2MBI5SA. The characteristic bands of 2MBI5SA are summarized in Supplementary Table S1; the molecular contains functional groups such as –SO_3_^−^, C–S, S–H, C=N, and C–N. For untreated chalcopyrite, the bands at 522.71 cm^−1^ can be assigned to Cu-S and Fe-S lattice vibrations, while the broad band at 3425.57 cm^−1^ is attributed to O-H stretching of adsorbed water or surface hydroxyls.

After treatment with 2MBI5SA, pronounced spectral changes were observed. The intensified band at 3434.84 cm^−1^ may be associated with N–H stretching and overlapping contributions from adsorbed water, suggesting involvement of the nitrogen-containing heterocycle. A band at 2827.60 cm^−1^ (possibly related to C–H stretching) and bands at 1592.36 cm^−1^ and 1356.46 cm^−1^, attributable to the C=N or C–N skeletal vibrations of the midazole or benzimidazole moiety, further support the interaction of 2MBI5SA with the chalcopyrite surface. A new band at 773.49 cm^−1^ may correspond to aromatic or heterocyclic C–H bending or possible C–S stretching.

Notably, the S–H band at 2578.83 cm^−1^ disappeared after treatment, suggesting deprotonation of the mercapto and its interaction with surface metal sites, consistent with stronger chemisorption. Moreover, red shifts of 2MBI5SA-related bands are likely associated with coordination-induced changes in the bonding environment or possible electron redistribution. Overall, these spectral features confirm the adsorption of 2MBI5SA on the chalcopyrite surface.

### 2.8. XPS Analysis

**Figure 9:**
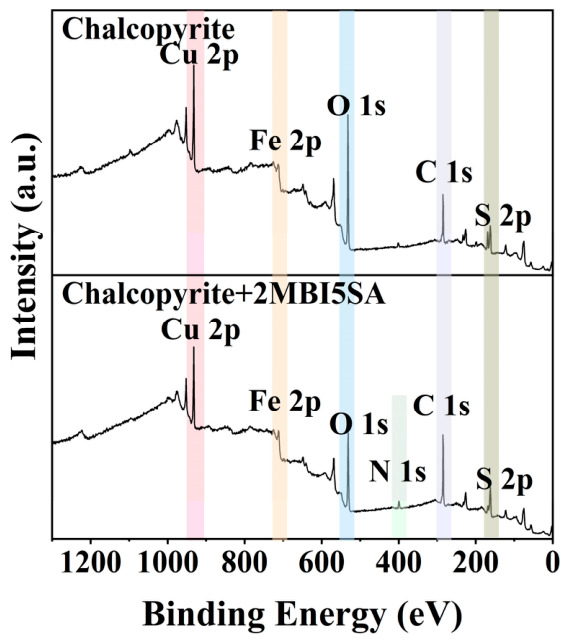
XPS spectra of chalcopyrite samples before and after 2MBI5SA treatment.

To further clarify the adsorption behavior of 2MBI5SA on chalcopyrite X-ray photoelectron spectroscopy (XPS) was performed on samples before and after conditioning with 2MBI5SA. The XPS survey spectra are presented in Figure 9. After treatment, a distinct N 1s signal appeared on the chalcopyrite surface, together with increased C 1s and O 1s signal intensities, indicating adsorption of the nitrogen-containing organic reagent. In addition, the surface elemental composition changed noticeably: the atomic percentages of Cu, Fe, O, and S decreased by 2.14%, 0.74%, 9.12%, and 2.7%, respectively, whereas the atomic percentage of carbon increased by 13.7%. These results suggest substantial surface coverage by 2MBI5SA and the formation of an organic layer on chalcopyrite. Meanwhile, no pronounced change in the Cu 2p and S 2p core-level features were observed, suggesting that the chalcopyrite sulfide lattice remained largely intact upon adsorption.

**Figure 10:**
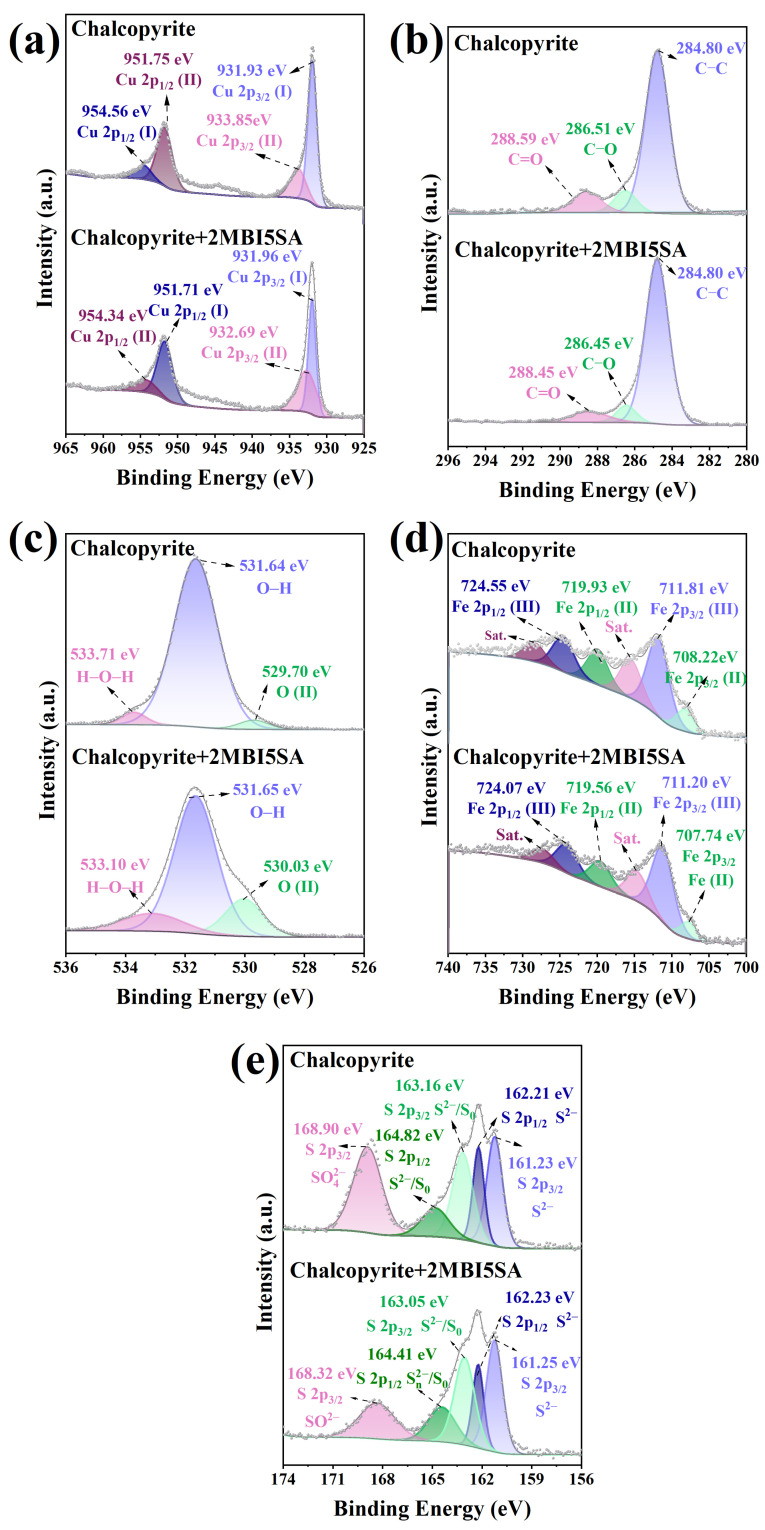
XPS spectra of 2MBI5SA, treated and untreated chalcopyrite samples by 2MBI5SA: (**a**) Cu 2p; (**b**) C 1s; (**c**) O 1s; (**d**) Fe 2p; (**e**) S 2p.

Figure 10a shows the high-resolution Cu 2p spectra of chalcopyrite before and after 2MBI5SA conditioning with 2MBI5SA. For the untreated sample, the Cu 2p region exhibits typical spin-orbit doublets. The peaks at 931.93 eV and 933.85 eV can be assigned to Cu (I) and Cu (II) in the Cu 2p_3/2_ component, while those at 951.75 eV and 954.56 eV correspond to Cu (I) and Cu (II) in Cu 2p_1/2_, respectively. After treatment with 2MBI5SA, the corresponding peaks shift to 931.96 eV, 932.69 eV, 954.34 eV, and 951.71 eV, respectively. The fitted-component atomic concentrations further showed that the Cu (I) and Cu (II) components in Cu 2p_1/2_ changed from 0.64% and 2.65% to 0.26% and 2.55%, respectively, while the Cu (II) and Cu (I) components in Cu 2p_3/2_ changed from 1.33% and 3.70% to 1.76% and 1.99%, respectively. The overall decrease in Cu (I)-related components and the relatively small variation in Cu (II)-related components indicate that the local chemical environment of Cu sites was modified after 2MBI5SA adsorption, without the formation of new dominant Cu species.

Figure 10b shows the C 1s spectra. In untreated chalcopyrite, the peaks at 284.80 eV, 286.51 eV, and 288.59 eV are attributed to C–C, C–O, and C–O species, respectively [60]. After 2MBI5SA adsorption, the C–O and C=O components shifted to 286.45 eV and 288.45 eV, respectively. The fitted-component atomic concentrations further showed that the C=O, C–O, and C–C components increased from 3.79%, 3.04%, and 23.70% to 3.88%, 3.23%, and 36.61%, respectively. The pronounced increase in the C–C component indicates a significant increase in organic carbon species on the chalcopyrite surface after 2MBI5SA treatment, which is consistent with the formation of an adsorbed organic reagent layer.

The O 1s spectra are shown in Figure 10c. In the untreated sample, peaks at 529.70 eV, 531.64 eV, and 533.71 eV are assigned to O (II) (oxide or lattice oxygen), O–H (hydroxyl species), and H–O–H (adsorbed water), respectively, suggesting the presence of surface oxidation products and hydration species. After treatment with 2MBI5SA, the O^2−^ and H–O–H components shifted to 530.03 eV and 533.10 eV, corresponding to changes of +0.33 eV and −0.61 eV, respectively. The fitted-component atomic concentrations further showed that the H–O–H component decreased from 30.88% to 3.01%, corresponding to a decrease of 27.87 percentage points, whereas the O–H and O^2−^ components increased from 1.29% and 1.12% to 16.14% and 3.92%, respectively. These variations indicate a pronounced redistribution of oxygen-containing surface species after 2MBI5SA adsorption, further suggesting that the local bonding environment and surface hydration or oxidation state of chalcopyrite were modified by reagent adsorption.

Figure 10d shows the Fe 2p spectra of chalcopyrite before and after reagent treatment with 2MBI5SA. In the untreated sample, the Fe 2p_3/2_ components at 708.22 eV and 711.81 eV are assigned to Fe (II) and Fe (III), respectively, while the Fe 2p_1/2_ components at 719.93 eV and 724.55 eV correspond to Fe (II) and Fe (III). After adsorption of 2MBI5SA, the Fe (II) and Fe (III) components of Fe 2p_3/2_ were located at 707.74 eV and 711.20 eV, respectively, whereas those of Fe 2p_1/2_ appeared at 719.56 eV and 724.07 eV, respectively. Relative to the untreated sample, these four peaks exhibited negative shifts of 0.48 eV, 0.61 eV, 0.37 eV, and 0.48 eV, respectively. The fitted-component atomic concentrations of Fe 2p showed that the Fe (III) and Fe (II) components in Fe 2p_1_/_2_ decreased from 1.03% and 1.01% to 0.86% and 0.76%, respectively, while the Fe (III) and Fe (II) components in Fe 2p_3_/_2_ decreased from 2.28% and 0.53% to 2.22% and 0.38%, respectively. The overall decrease in these Fe components may be related to the coverage of surface Fe sites by the adsorbed 2MBI5SA layer, and the binding-energy shifts further suggest that the local electronic environment around Fe sites was affected by reagent adsorption.

Figure 10e depicts the S 2p spectra of chalcopyrite before and after 2MBI5SA treatment. Three characteristic sulfur species were identified in the untreated chalcopyrite sample: S^2−^ (S 2p_3/2_ at 161.23 eV and S 2p_1/2_ at 162.21 eV), S_n_^2−^/S^0^ (S 2p_3/2_ at 163.16 eV and S 2p_1/2_ at 164.82 eV), and SO_4_^2−^ (S 2p_3/2_ at 168.90 eV). After adsorption of 2MBI5SA, the S^2−^ peaks showed only slight changes to 161.25 eV and 162.23 eV. The S_n_^2−^/S^0^ peaks shifted to 163.05 eV and 164.41 eV, while the SO_4_^2−^ component moved to 168.32 eV, corresponding to binding-energy decreases of 0.11 eV, 0.41 eV, and 0.58 eV, respectively. No new sulfur-related components were observed. The fitted-component atomic concentrations further showed that the SO_4_^2−^ component decreased from 5.73% to 2.86%, corresponding to a decrease of 2.87 percentage points. The S_n_^2−^/S^0^ components changed only slightly, with the S 2p_3/2_ component decreasing from 4.14% to 3.91% and the S 2p_1/2_component increasing from 1.83% to 2.09%. Meanwhile, the S^2−^ components remained relatively stable, with the S 2p_3/2_ component changing from 4.07% to 4.03% and the S 2p_1/2_ component decreasing from 2.53% to 2.08%. These results indicate that the sulfide lattice of chalcopyrite was largely preserved after 2MBI5SA adsorption, while the surface oxidized sulfur species and local electronic environment were partially altered.

Taken together, the XPS results indicate that adsorption of 2MBI5SA on chalcopyrite is associated with interactions between its S- and N-containing functionalities and surface Cu or Fe sites. The systematic changes observed in both the survey spectra and high-resolution spectra suggest that adsorption is accompanied by interfacial electronic redistribution and consistent with the formation of an adsorbed organic layer on the chalcopyrite surface. In addition, the shifts observed in the S 2p spectra imply that surface sulfur species also experience partial electronic perturbation upon 2MBI5SA adsorption, suggesting their involvement in interfacial interactions.

### 2.9. SEM-EDS Analysis

**Figure 11:**
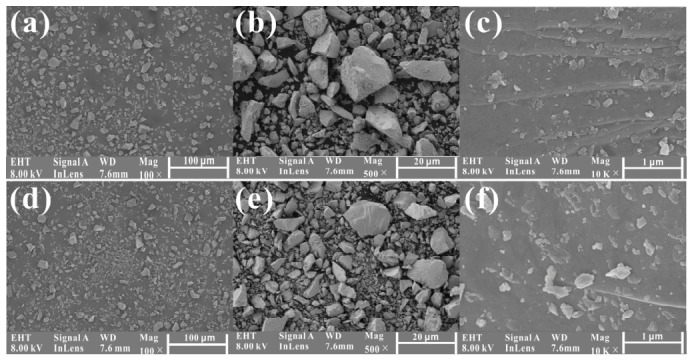
SEM images of untreated chalcopyrite at 100 μm (**a**), 20 μm (**b**), and 1 μm (**c**) and of treated chalcopyrite by 2MBI5SA at 100 μm (**d**), 20 μm (**e**), and 1 μm (**f**).

Figure 11 presents representative SEM images of chalcopyrite before and after 2MBI5SA treatment. Compared with the untreated sample, the treated chalcopyrite surface showed observable changes in surface texture and local particle aggregation. It should be noted that SEM images provide qualitative morphological information and should not be used alone as direct evidence for reagent adsorption. Therefore, the SEM observations are interpreted here only as supplementary evidence and are discussed together with the semi-quantitative EDS results and other surface analyses.

**Figure 12:**
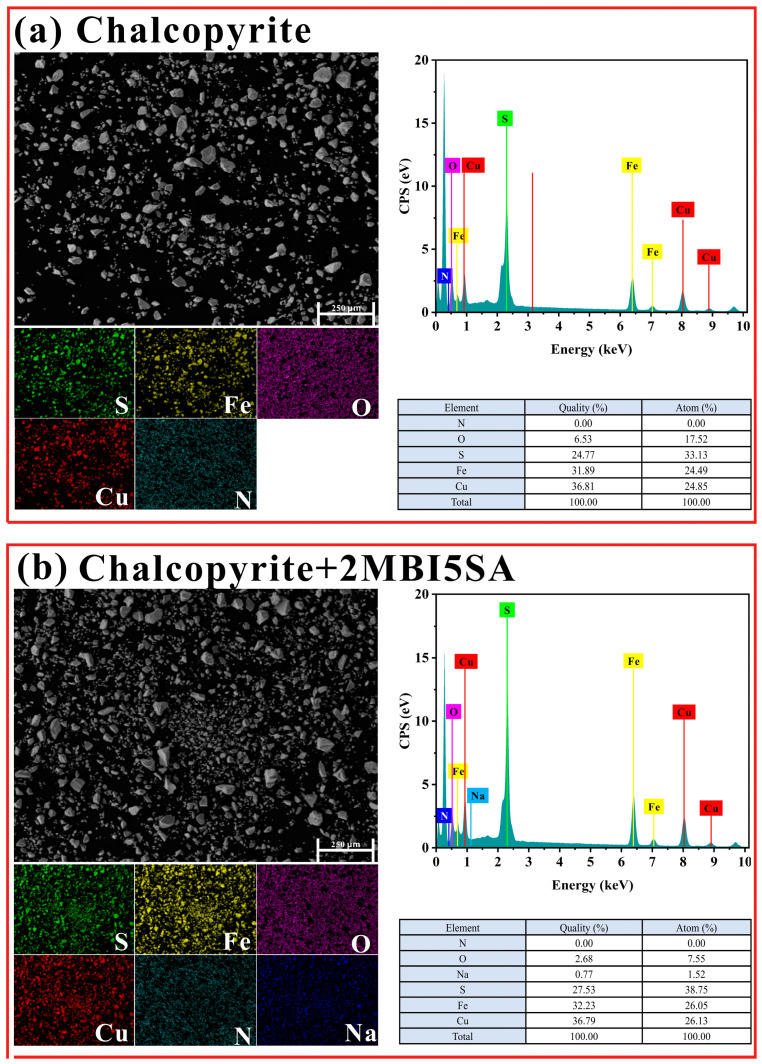
SEM-EDS surface compositional analysis of chalcopyrite before and after treatment with 2MBI5SA.

The EDS results in Figure 12 show changes in the surface elemental composition of chalcopyrite after 2MBI5SA treatment. In the untreated sample, the EDS-derived atomic percentages of O, S, Fe, and Cu were 17.52%, 33.13%, 24.49%, and 24.85%, respectively. After treatment, the O content decreased to 7.55%, whereas S, Fe, and Cu increased to 38.75%, 26.05%, and 26.13%, respectively. In addition, a small amount of Na was detected after 2MBI5SA treatment. These EDS results suggest that the surface composition of chalcopyrite was altered after reagent treatment. Together with the contact angle, FTIR, XPS, and SCC-DFTB results, the SEM-EDS analysis provides supporting evidence for the adsorption of 2MBI5SA on the chalcopyrite surface. 

### 2.10. Density Functional Theory (DFT) Calculations

#### 2.10.1. Electronic Structure Characteristics of the Reagent

**Figure 13:**
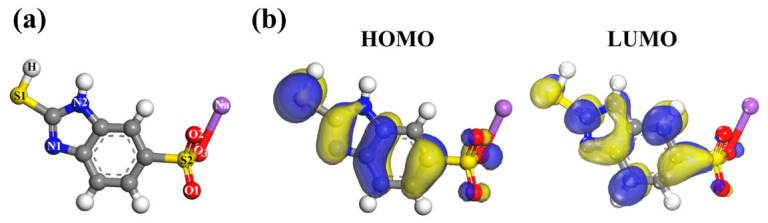
Molecular structure (**a**) and schematic of frontier molecular orbitals (**b**) of 2MBI5SA.

The chemical reactivity of a molecule is largely governed by its electronic structure; in particular, the highest occupied molecular orbital (HOMO) and the lowest unoccupied molecular orbital (LUMO) play a crucial role in influencing intermolecular interactions [61,62]. Accordingly, analysis of frontier molecular orbitals (FMOs) provides valuable insights into the adsorption propensity of flotation depressants on mineral surfaces. The optimized geometry and frontier-orbital distributions of 2MBI5SA are shown in Figure 13. The calculated HOMO is mainly localized on the S1 and N1 atoms and exhibits pronounced π-type character, suggesting that these atoms have strong electron-donating capability. These electronic features imply that S1 and N1 are probable electron-donor sites for interacting with surface metal centers. Meanwhile, the LUMO also exhibits appreciable contributions in the same region, indicating that these sites may also participate in electron acceptance during interfacial interactions. On chalcopyrite, surface Cu or Fe sites are generally Lewis-acidic and possess d orbitals that can overlap with the π-type orbitals of organic ligands, enabling orbital hybridization at the interface. Such electronic compatibility can facilitate interfacial charge transfer (or charge redistribution) between the reagent and the mineral surface.

**Figure 14:**
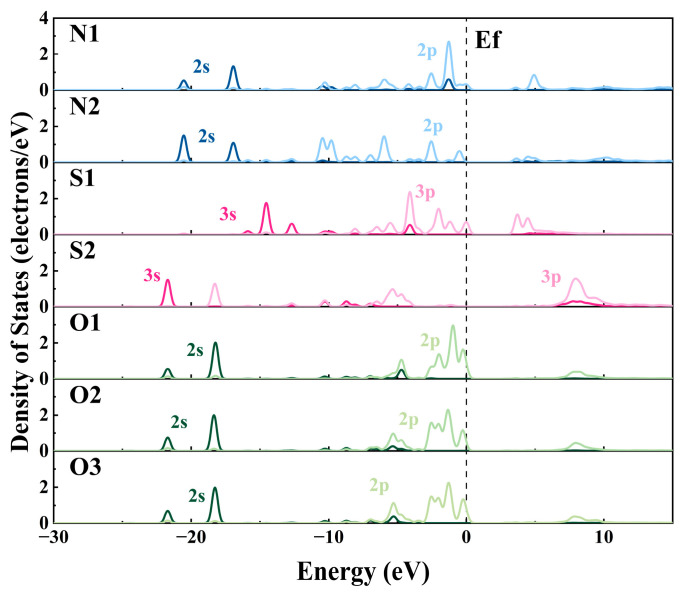
Partial density of states (PDOS) plot of 2MBI5SA.

To further evaluate the electronic activity of individual atoms, the partial density of states (PDOS) of key atoms in 2MBI5SA was analyzed (Figure 14). The results show that the N1 2p and S1 3p orbitals contribute substantially in the frontier-energy region (near Ef, the HOMO-LUMO region), suggesting that these sites are electronically active and may participate in interfacial charge transfer with the mineral surface. By contrast, the N2 2p and S2 3p states are mainly distributed at deeper energies and show negligible contributions near Ef, implying lower electronic activity. Although the O1, O2, and O3 2p states also contribute near the frontier-energy region, their intensities are comparatively low and are dominated by deeper-lying states, suggesting that they may play a secondary role in interfacial charge transfer. Overall, these PDOS results indicate that N1 and S1 are the most electronically active sites in 2MBI5SA and are therefore likely to contribute to its interaction with the chalcopyrite surface.

The reactive sites were further evaluated based on Mulliken charge analysis and condensed Fukui functions (Table 1). The calculated Mulliken charges indicate that N1, N2, S1, and the O atoms in 2MBI5SA carry negative charge, making these sites favorable for interaction with positively charged surface metal centers on chalcopyrite. The Fukui analysis shows that S1, N1, O1, and O2 have relatively large nucleophilic Fukui indices, with S1 exhibiting the highest electron-donating ability, followed by N1. Together, these results suggest that S1 and N1 are the primary reactive sites and likely play key roles in adsorption on the chalcopyrite surface.

#### 2.10.2. Adsorption Behavior of the Reagent on the Chalcopyrite Surface

**Figure 15:**
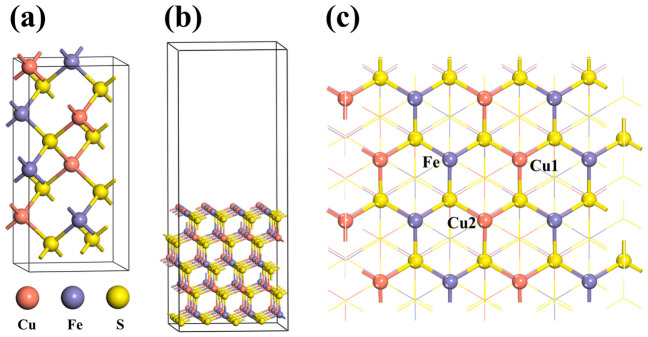
(**a**) Chalcopyrite unit cell model; (**b**) (112) surface model; (**c**) schematic illustration of adsorption sites on the chalcopyrite (112) surface.

To elucidate the adsorption mechanism of 2MBI5SA on chalcopyrite, self-consistent charge density-functional tight-binding (SCC-DFTB) calculations were performed. The optimized chalcopyrite (112) surface slab model is shown in Figure 15. The slab comprises five atomic layers built from a (2 × 2 × 1) supercell, and a 20 Å vacuum region was introduced to minimize interactions between periodic images. Based on the structural characteristics of the chalcopyrite (112) surface, surface Cu1 or Cu2 and Fe site were selected as potential adsorption sites. Five adsorption configurations were constructed (Figure S1) to represent plausible binding modes of 2MBI5SA on the chalcopyrite surface, and the calculated adsorption energies are summarized in Table 2. All calculated adsorption energies are negative, indicating that adsorption of 2MBI5SA on chalcopyrite is exothermic and energetically favorable. Moreover, the bidentate configurations generally exhibit more negative adsorption energies than the monodentate ones, suggesting that multidentate coordination enhances interfacial binding. Among the examined structures, the bidentate configuration with S1–Cu1 and N1–Cu2 coordination shows the most negative adsorption energy, indicating that this binding mode is the most favorable.

**Figure 16:**
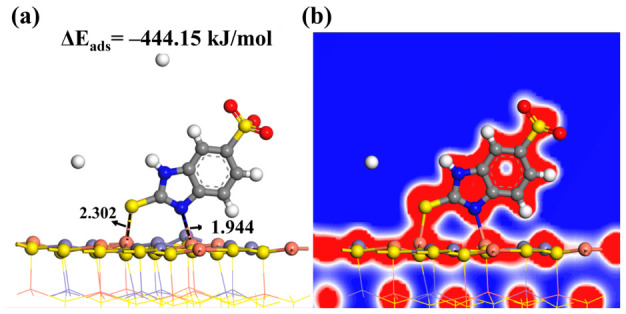
Adsorption of 2MBI5SA on the chalcopyrite surface: (**a**) adsorption model; (**b**) electron density map.

The optimized adsorption configuration is shown in Figure 16a. During the adsorption, the S1 site is assumed to deprotonate, and the resulting thiolate coordinates with a surface metal center. After adsorption, the S1–Cu1 and N1–Cu2 bond lengths are 2.302 Å and 2.344 Å, respectively, which are consistent with the formation of coordination bonds between 2MBI5SA and surface metal sites. The electron density map in Figure 16b shows clear electron-density overlap between S1–Cu1 and N1–Cu2, suggesting appreciable covalent character at the interface. The calculated adsorption energy is −444.15 kJ·mol^−1^, indicating highly exothermic adsorption, which can be attributed to bidentate coordination involving the mercapto (thiolate) group and the imine-like (C=N) nitrogen site.

**Figure 17:**
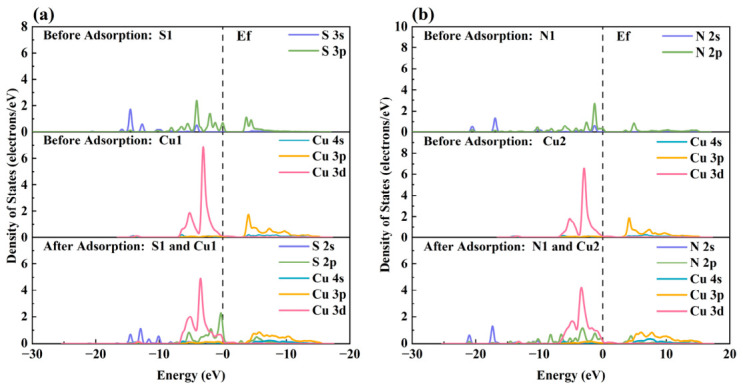
Partial density of states (PDOS) of interacting atoms before and after 2MBI5SA adsorption on chalcopyrite: (**a**) S and Cu1; (**b**) N1 and Cu2.

Figure 17 compares the PDOS of the key interfacial atoms before and after adsorption. After adsorption, the S1 3p and N1 2p states shift to lower energies and broaden in the -7 to 0 eV range, showing substantial overlap with the Cu 3d states. These features suggest pronounced orbital hybridization and interfacial charge redistribution. Meanwhile, the Cu 3d peaks decrease in intensity and broaden, implying enhanced electron delocalization, which may contribute to stabilizing the interfacial bonding. Notably, the hybridized states associated with S1–Cu bonding extend toward the Fermi level, whereas those related to N1–Cu remain below Ef, suggesting that the S–Cu interaction plays a larger role in interfacial charge transfer.

Mulliken charge analysis was further conducted to quantify charge redistribution upon adsorption (Table 3). The results indicate charge transfer from S1 to Cu1, along with partial electron-density redistribution from Cu2 toward N1, consistent with strong interfacial bonding and a pronounced chemisorption character.

**Figure 18:**
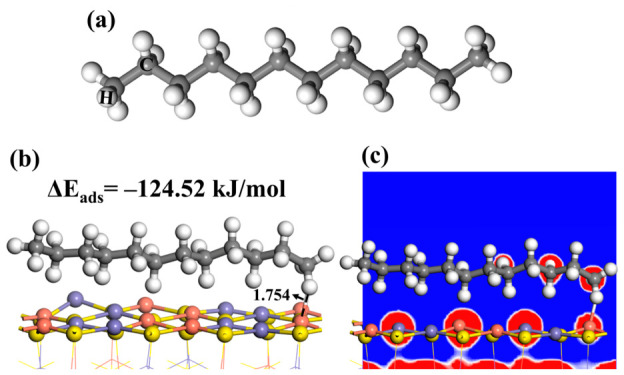
Molecular model of C_12_H_26_ (**a**) and its adsorption configurations on the chalcopyrite surface (**b**) and the electron density map (**c**).

Kerosene mainly consists of hydrocarbon mixtures predominantly in the C_12_–C_16_ range, with dodecane (C_12_H_26_) commonly used as a representative component [63,64]. Accordingly, C_12_H_26_ was employed as a kerosene surrogate in adsorption simulations to probe how 2MBI5SA influences chalcopyrite flotation recovery. The molecular structure of C_12_H_26_ is shown in Figure 18a, while the adsorption configuration and electron-density map are provided in Figure 18b,c. The electron-density map shows no new bond formation or appreciable electron-density overlap between C_12_H_26_ and the chalcopyrite surface, indicating that kerosene adsorption is dominated by physisorption rather than chemisorption [65]. Moreover, the calculated adsorption energy (−124.52 kJ·mol^−1^) indicates that, compared with 2MBI5SA, kerosene exhibits substantially weaker affinity for the chalcopyrite surface. Therefore, even in the presence of kerosene, 2MBI5SA is expected to remain effective in suppressing chalcopyrite flotation by outcompeting kerosene for surface sites.

### 2.11. Comparative Analysis of the Structure and Function Relationship

To further clarify the effect of introducing hydrophilic functional groups on reagent performance, a comparative analysis between the parent collector-type skeleton and the hydrophilic-group-modified reagent 2MBI5SA was added based on previously reported studies, molecular structural features, and the experimental results obtained in this work. It should be noted that the description of the parent collector-type skeleton in Table 4 is mainly based on the literature reports and structural analysis, whereas the discussion of 2MBI5SA is supported by the flotation, contact angle, zeta potential, FTIR, XPS, and SCC-DFTB results obtained in this study.

As shown in Table 4, 2MBI5SA does not simply inherit the collecting behavior of the parent collector-type skeleton. Instead, it retains the mineral-affinitive mercapto group and benzimidazole N atoms while introducing a hydrophilic and ionizable sulfonate group, thereby markedly changing the hydrophilic or hydrophobic balance and interfacial role of the molecule. On the one hand, the S and N sites provide the structural basis for selective coordination adsorption on the chalcopyrite surface. On the other hand, the sulfonate group enhances molecular hydrophilicity, ionization ability, and surface hydration, thereby increasing the hydrophilicity of chalcopyrite and suppressing the further adsorption and spreading of kerosene on the chalcopyrite surface. Therefore, the structural modification effect of 2MBI5SA can be summarized as the synergistic action of selective coordination adsorption sites and the hydrophilic sulfonate group, which converts a collector-type molecular skeleton from a flotation-enhancing function into a selective chalcopyrite-depressing function.

## 3. Experiments

### 3.1. Materials and Depressants

Chalcopyrite and molybdenite samples used for microflotation tests were obtained from a mining site in Panzhihua, Sichuan Province, and another mining site in Jiangxi Province, China, respectively. The two ores were subjected to crushing, hand sorting, gangue removal, ceramic ball milling, and sieving to produce particle size fractions of −109+58 µm and −58 µm. The −109+58 µm fraction was used for flotation tests and adsorption measurements, while the −58 µm fraction was further ground in an agate mortar to −2 µm for FTIR and XPS analyses. Phase identification and chemical composition of chalcopyrite and molybdenite were determined by X-ray diffraction using a Bruker D8 Advance diffractometer (BRUKER AXS GmbH, Karlsruhe, Germany) and X-ray fluorescence spectrometry using an S8 TIGER XRF analyzer (BRUKER, Germany), and the results are shown in Supplementary Figures S2 and S3. The purities of chalcopyrite and molybdenite were determined to be 99% and 96%, respectively, which meets the requirements of the present study.

Industrial Cu–Mo bulk concentrate samples for flotation tests were collected from the Dexing copper mine, a typical porphyry Cu–Mo deposit in Jiangxi Province, China. The Cu–Mo bulk concentrate was freshly collected on site from the Dexing concentrator, immediately packed in sealed bags, vacuum-sealed to minimize air exposure and stored at low temperature before use to reduce surface oxidation. The sample used for particle-size analysis was taken directly from the as-received Cu–Mo bulk concentrate after homogenization and quartering, without any additional grinding, regrinding, or screening after grinding. X-ray diffraction analysis of the bulk concentrate identified chalcopyrite, pyrite, enargite, and molybdenite as the main metallic minerals, with quartz and, minor muscovite as the main gangue minerals. The XRD patterns and particle size distribution are presented in Supplementary Figure S4, and its multi-element chemical composition is listed in Table 5.

Hydrochloric acid and sodium hydroxide used for pH adjustment were purchased from Chengdu Cologne Chemical Co., Ltd, Chengdu, China. The depressant 2-mercapto-5-benzimidazole sulfonate dihydrate and frother methyl isobutyl carbinol (MIBC) were obtained from Shanghai Aladdin Biochemical Technology Co., Ltd., Shanghai, China, while kerosene was obtained from Guangdong Linshi Chemical Depressants Co., Ltd, Huizhou, Guangdong, China. MIBC was of industrial grade, whereas all other depressants were of analytical grade. Deionized water was used in all experiments and analytical measurements. Deionized water was used to minimize the interference of dissolved ions and residual reagents, thereby allowing the intrinsic depression behavior and adsorption mechanism of 2MBI5SA to be evaluated under controlled laboratory conditions.

### 3.2. Microflotation Tests

Single-mineral and synthetic bulk-mineral flotation tests were conducted using an XFGC II air-sparged flotation cell (40 mL) with a constant agitation speed of 1740 rpm. Before flotation, 2.0 g of mineral sample was placed in a beaker containing 40 mL of deionized water, ultrasonically dispersed for 2 min and then allowed to settle for 2 min; the supernatant containing readily suspended fines was discarded. This step was used as a standardized pre-cleaning procedure to reduce the influence of suspended fines, rather than as a complete desliming process. The resulting slurry was transferred to the flotation cell and pre-agitated for 1 min. pH modifiers, depressants, collectors, and frothers were then added sequentially, and the pulp was conditioned for 1 min, 3 min, 3 min, and 2 min after each addition, respectively. Thereafter, air was introduced and froth collection was initiated; froth products were collected at 10 s intervals over a total flotation time of 3 min. After flotation, froth products and tailings were filtered, dried and weighed to calculate recovery. All single-mineral and synthetic mixed-mineral flotation tests were performed in triplicate, and the results are presented as the mean ± standard deviation.

For synthetic mixed-mineral flotation tests, a synthetic mixture of chalcopyrite and molybdenite at a mass ratio of 1:1 was used, and the flotation procedure was the same as that of the single-mineral tests. The flotation recoveries of single-mineral tests were calculated from the masses of the froth and tailing products, while those of synthetic mixed-mineral tests were calculated by mass balance using product masses and Cu or Mo grades. The complete flotation procedure is shown in Supplementary Figure S5.

### 3.3. Adsorption Capacity Measurement

The adsorption capacity of 2MBI5SA on the mineral surface was quantified using a Shimadzu UV-2450 ultraviolet-visible spectrophotometer (Shimadzu Corporation, Kyoto, Japan). The characteristic absorption peak of 2MBI5SA occurs at a wavelength of 240 nm. A series of standard solutions of 2MBI5SA were prepared, and their absorbance at 240 nm was measured to construct a calibration curve. Adsorption tests were conducted at 25 °C and pH 7: 0.50 g sample of ultrasonically cleaned chalcopyrite or molybdenite (−109+58 µm) was bulk with 40 mL of deionized water and stirred for 1 min. After adding 2MBI5SA at predetermined concentrations, the suspension was magnetically stirred for 10 min until adsorption equilibrium was reached. The mixture was then filtered through a 0.45 µm membrane, and the absorbance of the filtrate at 240 nm was measured to determine the residual concentration of 2MBI5SA. The adsorption amount was calculated using Equation (1). All tests were performed in triplicate, and the results were reported as average values.

(1) $$Q\;=\frac{{(C}_{i}-C_{s})V}{m}\;\times \;100\%$$

In the equation, Q denotes the amount of reagent adsorbed on the mineral surface at equilibrium (mg/g); $C_{i}$ and $C_{s}$ represent the initial reagent concentration and the equilibrium concentration of the reagent in the supernatant after reaction (mg/L), respectively; V is the solution volume (mL); and m is the mass of the mineral sample (g).

### 3.4. Contact Angle Measurement

To assess changes in surface wettability of the minerals before and after 2MBI5SA treatment, the contact angles of chalcopyrite and molybdenite under different treatment were measured using a DSA100E optical video contact angle goniometer (KRÜSS GmbH, Hamburg, Germany). High-purity chalcopyrite and molybdenite blocks were cut into specimens of approximately 2 cm × 2 cm × 2 cm and sequentially polished with 200, 500, and 2000 grit sandpapers to obtain smooth surfaces. The specimens were then ultrasonically cleaned in deionized water and vacuum-dried at 30 °C prior to testing.

Reagent-treated samples were prepared as follows: the specimens were immersed in a 2MBI5SA solution (1 × 10^−3^ mol/L, pH 7) for 15 min; and one set of specimens was subsequently immersed in a collector solution for an additional 10 min. After treatment, all samples were rinsed three times with deionized water and vacuum-dried before analysis. Control specimens were prepared using the same procedure but without reagent treatment.

### 3.5. Zeta Potential Measurement

The zeta potentials of chalcopyrite and molybdenite before and after reagent treatment were measured at 25 °C using a multi-angle particle size and high-sensitivity zeta potential analyzer (Brookhaven Instruments, Holtsville, NY, USA) to evaluate the effects of reagent adsorption [66]. In each test, 2.0 g of ultrasonically cleaned chalcopyrite or molybdenite powder was added to 40 mL of deionized water and magnetically stirred for 15 min, after which the slurry pH was adjusted to the target value. The suspension was allowed to stand for 10 min to allow the solid to settle, and the supernatant was collected with a syringe and transferred into disposable centrifuge tubes for zeta potential analysis.

### 3.6. Fourier Transform Infrared (FTIR) Spectroscopy

The Fourier transform infrared spectra of chalcopyrite before and after 2MBI5SA treatment were recorded using an IRTracer-100 spectrometer (Shimadzu Corporation, Kyoto, Japan). Samples were prepared by the KBr pellet method. Spectra were collected at a resolution of 4 cm^−1^ over the range of 400–4000 cm^−1^, with 32 co-added scans per sample. To minimize scattering effects, only mineral powders with a particle size of <2 µm were used. For sample preparation, 2.0 g of chalcopyrite was bulk with 40 mL of deionized water containing 2MBI5SA at a predetermined concentration in the flotation cell and stirred for 30 min to allow reagent adsorption. The treated slurry was then filtered, rinsed at least three times with deionized water and dried in a vacuum oven at 30 °C for 24 h prior to measurement.

### 3.7. X-Ray Photoelectron Spectroscopy (XPS)

The surface chemical compositions of chalcopyrite before and after 2MBI5SA treatment was analyzed by X-ray photoelectron spectroscopy using a Thermo Fisher Scientific K-Alpha instrument (Thermo Fisher Scientific, Waltham, MA, USA). For sample preparation, 2.0 g of chalcopyrite was ultrasonically pretreated for 3 min, then bulk with 40 mL of deionized water containing 2MBI5SA at a specified concentration in the flotation cell and magnetically stirred for 30 min. The slurry was subsequently filtered, and the recovered solids were rinsed three times with deionized water and dried in a vacuum oven at 30 °C prior to XPS analysis.

### 3.8. Scanning Electron Microscopy with Energy-Dispersive Spectroscopy (SEM-EDS)

For morphological and elemental analysis, treated and untreated chalcopyrite samples were affixed directly onto conductive adhesive and examined using a Sigma 300 scanning electron microscope (Carl Zeiss AG, Oberkochen, Germany) at an accelerating voltage of 8 kV to observe surface morphology changes induced by 2MBI5SA treatment. In addition, elemental distributions were characterized by energy-dispersive X-ray spectroscopy (EDS) in area scan mode at an accelerating voltage of 20 kV to investigate changes in surface chemical composition.

### 3.9. SCC-DFTB Computational Modeling

Quantum chemical calculations have become an important approach for elucidating the adsorption mechanisms of depressants on mineral surfaces [67,68]. The self-consistent charge density functional tight-binding (SCC-DFTB) method, as an approximate quantum mechanical technique that balances computational efficiency and accuracy, accelerates calculations by approximately two orders of magnitude compared with conventional density functional theory (DFT). Moreover, it has been shown to yield adsorption configurations, bond lengths, and Mulliken charge distributions that are in strong agreement with results from high-accuracy DFT for large sulfide systems [69,70,71,72].

In this study, the adsorption of 2MBI5SA on the chalcopyrite surface was systematically investigated using the SCC-DFTB method as implemented in Materials Studio 2019 (MS). All calculations were performed without spin restriction and under symmetry constraints. Geometry optimizations were carried out using the DFTB+ program, employing the CuFeOrg Slater–Koster parameter set which includes Cu, Fe, C, H, O, N, and S elements [70,72]. The experimental unit-cell parameters of chalcopyrite are a = b = 5.290 Å, c = 10.422 Å, and the optimized values from DFTB+ were a = 5.1147 Å, b = 5.1137 Å, c = 10.4083 Å [73]. Verification based on Equation (S1) in Supplementary Materials [74] showed a deviation of only 0.07% from the experiment values, confirming the reliability of the selected method and parameter set. The thermodynamically most stable chalcopyrite (112) surface was selected for modeling, as supported by interatomic potential analyses and literature DFT results [75]. The surface energies are detailed in Supplementary Equation (S2) and Table S2. A 2 × 1 × 1 supercell model was constructed, consisting of five atomic layers and a 20 Å vacuum gap. To mimic bulk constraints, the bottom three atomic layers were fixed during optimization, while the top two layers and adsorbate molecule were allowed to relax. The self-consistent field (SCF) convergence criterion was set at 1 × 10^−5^ eV/atom, with medium precision and a 1 × 2 × 1 k-point grid. Adsorption energies $E_{ads}$ were calculated according to Equation (2).

(2) $$E_{\mathrm{ads}}={\;E}_{\mathrm{adsorbate}/\mathrm{slab}}\;-\;(E_{\mathrm{adsorbate}}+{\;E}_{\mathrm{slab}})$$

where $E_{\mathrm{adsorbate}/\mathrm{slab}}$ represents the total energy of the reagent molecule adsorbed on the chalcopyrite surface, and $E_{\mathrm{adsorbate}}$ denotes the total energy of the optimized isolated reagent molecule. A more negative $E_{\mathrm{ads}}$ value indicates a stronger interaction between the reagent and the chalcopyrite surface.

To further evaluate the adsorption strength and bonding nature, density of states (DOS), charge density, and Mulliken charge analyses were performed using the CASTEP module. Calculations were carried out within the generalized gradient approximation (GGA) using the PW91 exchange-correlation functional, with a plane-wave cutoff energy of 400 eV. The self-consistent field (SCF) convergence criterion was set to 2.0 × 10^−5^ eV/atom.

It should be noted that the present SCC-DFTB calculations were performed using an idealized vacuum slab model. This setup was mainly used to identify the intrinsic adsorption tendency and possible coordination sites of 2MBI5SA on the chalcopyrite surface, and it did not explicitly include solvation effects, pH-dependent protonation or deprotonation equilibria, or competitive adsorption of water molecules.

### 3.10. Laboratory Flotation Tests on Actual Cu–Mo Bulk Concentrate

Laboratory flotation tests on actual Cu–Mo bulk concentrate samples were conducted using a 0.75 L XFD flotation machine (Jilin Prospecting Machinery Factory, Changchun, China). The Cu–Mo bulk concentrate was used directly without regrinding because its particle-size distribution indicated that the sample had already reached a relatively fine size range, with the −74 μm fraction accounting for 78.82% and containing 85.56% of Mo and 84.40% of Cu. Further regrinding could increase the proportion of ultrafine or slime particles, resulting in slime coating, higher reagent consumption, and reduced flotation selectivity; therefore, no regrinding was conducted before the actual ore flotation tests. For each test, 340 g of ore was used, and the impeller speed was maintained at 1992 rpm. After conditioning with the depressant only (without collector or frother) for 3 min, flotation was carried out for an additional 3 min. Finally, the resulting concentrate and tailings were filtered, dried and analyzed to determine Cu and Mo grades and recoveries. Each actual Cu–Mo bulk concentrate flotation test was conducted in triplicate under identical conditions, and the results are reported as the mean ± standard deviation.

## 4. Conclusions

This study is the first to report the application of 2-mercapto-5-benzoimidazole sulfonate dihydrate (2MBI5SA) as a chalcopyrite depressant in the flotation separation of Cu–Mo bulk concentrate flotation separation. The results indicate that 2MBI5SA can selectively depress chalcopyrite flotation by forming stable chemisorbed complexes with surface Cu sites via its –SH and C=N functional groups. The work also confirms that introducing hydrophilic and ionizable functional groups into a collector framework can convert a collector from a flotation promoter into a selective depressant, thereby enabling targeted suppression of specific minerals. This study provides a foundation for the development of effective chalcopyrite depressants for Cu–Mo flotation separation.

(1) Contact angle measurements showed that 2MBI5SA markedly increased the hydrophilicity of the chalcopyrite surface while having minimal influence on molybdenite. Furthermore, after 2MBI5SA adsorption on the chalcopyrite surface, subsequent kerosene treatment had little effect on the contact angle, suggesting that 2MBI5SA adsorption effectively suppress kerosene adsorption and spreading on chalcopyrite. In contrast, for molybdenite, pre-adsorption of 2MBI5SA followed by kerosene treatment caused negligible change. Zeta potential analysis further supported these observations. Microflotation tests confirmed that over a pH range of 7–13, 2MBI5SA effectively suppressed chalcopyrite flotation while exerting only a minor effect on molybdenite recovery, demonstrating good selectivity.
(2) Industrial flotation tests of actual Cu–Mo bulk concentrate revealed that, compared with the conventional depressant sodium sulfide (Na_2_S), 2MBI5SA exhibited stronger selective depression toward chalcopyrite. Under conditions where molybdenite recovery reached 81.46% and the Mo grade in the concentrate was 4.46%, chalcopyrite recovery decreased to 13.03%.
(3) SCC-DFTB calculations showed that 2MBI5SA adsorbs onto Cu sites on the chalcopyrite surface, forming stable adsorption configurations with Cu–heteroatom bond lengths of 2.302 Å and 2.344 Å, and an adsorption energy of −444.15 kJ·mol^−1^. In contrast, kerosene adsorption on the chalcopyrite surface is significantly weaker, indicating that 2MBI5SA can effectively suppress chalcopyrite flotation even in the presence of a collector. Although these SCC-DFTB simulations were performed under vacuum conditions, the trends obtained are consistent with experimental observations.

The toxicity, degradability, and environmental fate of 2MBI5SA have not yet been systematically evaluated, and these aspects will be further investigated in future work.

## Figures and Tables

**Table 1:** Calculated results of Mulliken charges and Fukui functions for the 2MBI5SA molecule.

Atomic	N1	N2	S1	S2	O1	O2	O3
Mulliken charges	−0.352	−0.242	−0.264	0.572	−0.493	−0.538	−0.531
Fukui(f^+^)	0.031	0.022	0.117	0.020	0.031	0.031	0.025

**Table 2:** Adsorption energy of 2MBI5SA on the different sites of the chalcopyrite (112) surface.

Adsorption Site	Adsorption Energy (kJ/mol)
S1–Cu1	−383.55
S1–Fe1	−331.54
S1–Fe1 and N1–Fe2	−381.24
S1–Cu1 and N1–Cu2	−444.15
S1–Cu1 and N1–Fe1	−404.84

**Table 3:** Mulliken charge of interacting atoms before and after 2MBI5SA adsorption.

Atom	Mulliken Charge		D-Value
	Before Adsorption	After Adsorption	
Cu1	0.27	0.20	−0.07
Cu2	0.26	0.34	0.08
S1	−0.26	−0.16	0.10
N1	−0.35	−0.42	−0.07

**Table 4:** Structural and functional comparison between the parent collector-type skeleton and the hydrophilic-group-modified reagent 2MBI5SA.

Comparison Item	2MBI	2MBI5SA
Molecular structure	Mainly composed of a hydrophobic skeleton and mineral-affinitive groups, which usually interact with surface metal sites of sulfide minerals through electron-donating atoms such as S and N.	Retaining the mercapto group and benzimidazole N atoms as mineral-affinitive sites, while introducing a strongly hydrophilic and ionizable sulfonate group.
Hydrophilic or hydrophobic balance	More inclined to enhance mineral surface hydrophobicity, thereby improving the floatability of target minerals.	Showing enhanced hydrophilicity and ionization ability; the sulfonate group can strengthen surface hydration and electrostatic repulsion.
Main function	Mainly exhibiting conventional collecting behavior by increasing mineral surface hydrophobicity and floatability through adsorption or coordination.	Mainly exhibiting selective depression and interfacial regulation by preferentially acting on chalcopyrite and reducing its floatability.
Evidence	Mainly supported by previous reports on 2MBI-type structures as chelating collectors or collector-type skeletons for sulfide minerals.	The results in this study demonstrate that 2MBI5SA selectively adsorbs on the chalcopyrite surface and depresses chalcopyrite through S or N-site coordination and sulfonate-induced hydrophilic regulation.

**Table 5:** Multi-element chemical composition analysis of the Cu–Mo bulk concentrate.

**Elements**	**Fe**	**S**	**Cu**	**Si**	**Al**	**K**	**Ca**	**Mg**
Content (%)	29.70	24.80	24.50	10.70	4.28	2.03	1.46	0.82
**Elements**	**Mo**	**Ti**	**Er**	**Zn**	**Pb**	**P**	**Other**	**–**
Content (%)	0.79	0.28	0.25	0.12	0.006	0.005	0.159	–

## Data Availability

The original contributions presented in this study are included in the article. Further inquiries can be directed to the corresponding author.

## Supplementary Materials

The following supporting information can be downloaded at: https://www.mdpi.com/article/10.3390/molecules31132383/s1, Figure S1: Adsorption configurations of 2MBI5SA on different sites of the chalcopyrite surface: (a) S1-Cu; (b) S1-Fe1; (c) S1-Fe1 and N1-Fe2; (d) S1-Cu1 and N1-Fe1; (e) S1-Cu1 and N1-Cu2. Figure S2: X-ray diffraction (XRD) patterns of the single minerals: (a) chalcopyrite and (b) molybdenite. Figure S3: Multi-element chemical composition analysis of the single minerals: (a) chalcopyrite and (b) molybdenite. Figure S4: XRD patterns (a) and particle size distribution results of the actual copper-molybdenum mixed concentrate sample. Figure S5: Flowchart of the micro-flotation experiments. Table S1: Major groups within 2MBI5SA molecule. Table S2: Surface energy measurement results of chalcopyrite.
