# Supplementary material for: Study on the Depression Performance and Mechanism of the Novel Chalcopyrite Depressant 2-Mercapto-5-benzimidazole Sulfonate Dihydrate in the Flotation Separation of Cu-Mo Bulk Concentrate
Source: Molecules. 2026 Jul 6;31(13):2383. doi: 10.3390/molecules31132383 (PMC13362721; doi:10.3390/molecules31132383)
Supplement: Supplementary file 1 [file molecules-31-02383-s001.zip › molecules-4342769-supplementary.pdf]

Supplementary material

2. Results and Discussion

2.7 FTIR Analysis

The characteristic bands of 2MBI5SA are summarized in Supplementary material Table S1

Table S1. Major groups within 2MBI5SA molecule.

Wavenumber(cm^{-1})	Groups
3585.60	O–H stretching
3503.06	imidazole N–H stretching
3277.81	aromatic or imidazole C–H asymmetric stretching
3051.33	aromatic or imidazole C–H asymmetric stretching
2578.83	S–H stretching
1654.85	imidazole C=N conjugated
1610.55	imidazole C=N conjugated
1517.95	aromatic C=C stretching
1475.50	aromatic C=C stretching
1382.98	imidazole C–N stretching
1334.73	imidazole C–N stretching
1203.65	S=O asymmetric stretching
1172.24	S=O asymmetric stretching
1082.09	S=O symmetric stretching
1023.27	S=O symmetric stretching
891.95	aromatic or imidazole C–H out-of-plane bending
823.60	aromatic or imidazole C–H out-of-plane bending
756.09	aromatic and imidazole ring C–H bending
678.96	C–S stretching
628.76	C–S stretching
526.56	S–O bending

2.10. Density Functional Theory (DFT) Calculations

2.10.2 Adsorption Behavior of the Reagent on the Chalcopyrite Surface

In this study, Cu1, Cu2, Fe1, and Fe2 sites on the (112) surface were selected as active adsorption sites, and five distinct adsorption configurations were calculated

(Figure S1) to simulate the adsorption behavior of 2MBI5SA at different sites on the chalcopyrite surface.

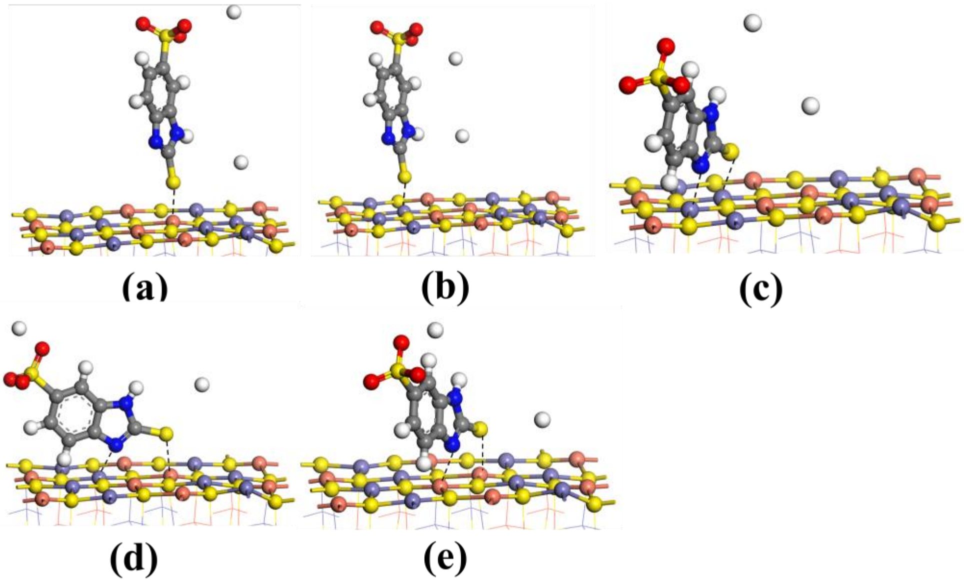

Figure S1. Adsorption configurations of 2MBI5SA on different sites of the chalcopyrite surface: (a) S1-Cu; (b) S1-Fe1; (c) S1-Fe1 and N1-Fe2; (d) S1-Cu1 and N1-Fe1; (e) S1-Cu1 and N1-Cu2.

3. Experiment

3.1 Materials and Depressants

The mineral compositions and chemical compositions of the chalcopyrite and molybdenite samples used in the microflotation experiments were characterized, and the results are presented in Figures S2 and S3, respectively. For the industrial ore flotation tests, the XRD patterns and particle size distribution of the actual mixed copper-molybdenum concentrate are shown in Figure S4.

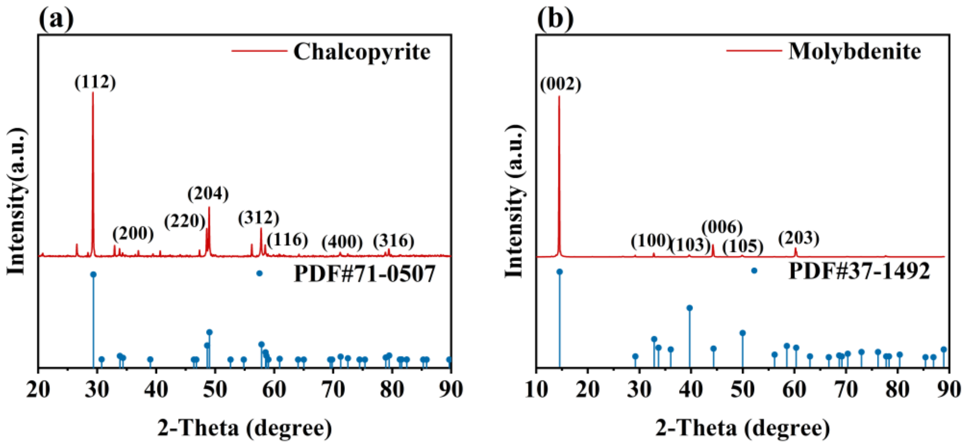

Figure S2. X-ray diffraction (XRD) patterns of the single minerals: (a) chalcopyrite and (b) molybdenite.

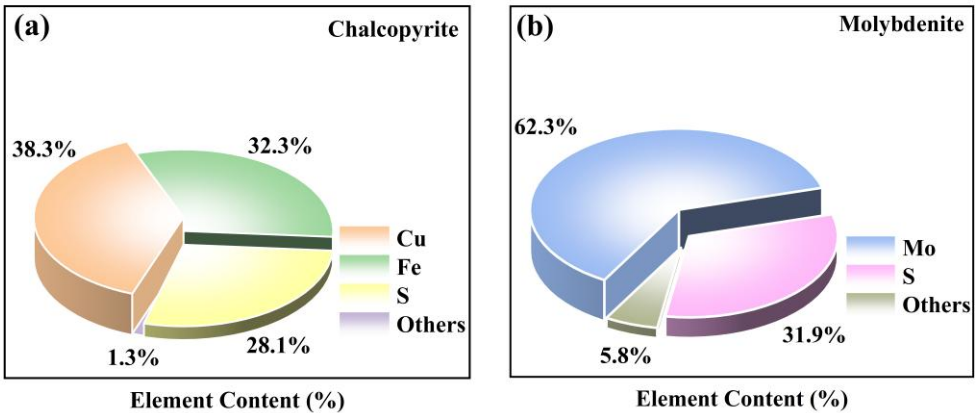

Figure S3. Multi-element chemical composition analysis of the single minerals: (a) chalcopyrite and (b) molybdenite.

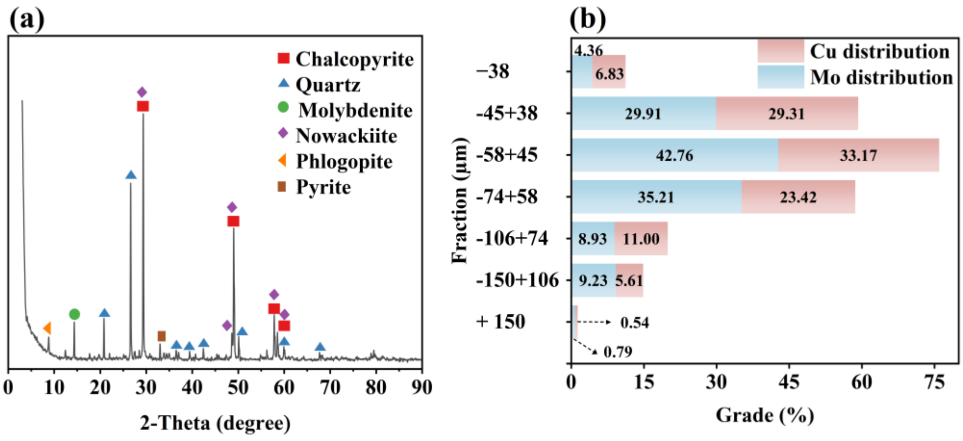

Figure S4. XRD patterns (a) and particle size distribution results of the actual copper-molybdenum mixed concentrate sample.

3.2 Microflotation Tests

The complete flotation procedure is illustrated in Supplementary Figure S5.

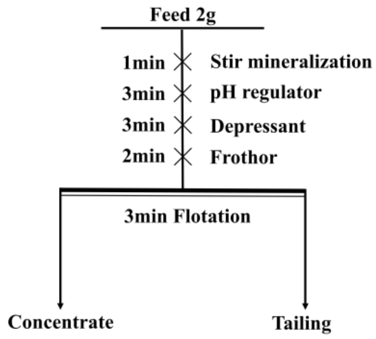

Figure S5. Flowchart of the micro-flotation experiments.

3.9 SCC-DFTB Computational Modeling

Using Equation (S1), the lattice parameters of the optimized chalcopyrite (112) surface obtained from the DFTB+ module were validated against experimental values to assess the reliability of the CuFeOrg Slater–Koster parameter set. The formula for calculating surface energy and the results are presented in Formula (S2) and Table S2. When the number of atomic layers reaches five, the surface energy tends towards stability; consequently, five atomic layers were selected as the computational benchmark.

Table S2. Surface energy measurement results of chalcopyrite.

Number of atomic layers	3	4	5	6	7
Surface Energy (J/m^2)	0.7779	0.8081	0.8158	0.8165	0.8178

$$D=\frac{\sqrt{(a\text{-}a')^2+(b\text{-}b')^2+(c\text{-}c')^2}}{3}\times 100\% \tag{S1}$$

$$E_{surf}=\frac{E_{slab}\text{-}nE_{bulk}}{2A} \tag{S2}$$

Here, D denotes the percentage error, while a , b and c represent the experimental values of the mineral unit cell parameters. a', b, and c' denote the theoretical values of the mineral unit cell parameters. E_{surf} denotes surface energy, E_{slab} represents the total energy of the mineral unit cell, E_{bulk} denotes the total energy of the optimised mineral unit cell, n indicates the ratio of surface atoms to total atoms in the unit cell, and $A=a\times b$ represents the surface area of the mineral unit cell.
